# Growth Promoting Activity of *Annona muricata* L. Leaf Extracts on *Lactobacillus casei*

**DOI:** 10.3390/plants11050581

**Published:** 2022-02-22

**Authors:** Nimcy Noemí Meza-Gutiérrez, Paola Magallón-Servín, Rosendo Balois-Morales, Iza Fernanda Pérez-Ramírez, Graciela Guadalupe López-Guzmán, Guillermo Berumen-Varela, Pedro Ulises Bautista-Rosales

**Affiliations:** 1 Programa de Doctorado en Ciencias Biológico Agropecuarias, Universidad Autónoma de Nayarit, Km 9 Carretera Tepic-Compostela, Xalisco C.P. 63180, Nayarit, Mexico; nimcy.meza@uan.edu.mx (N.N.M.-G.); rmbalois@uan.edu.mx (R.B.-M.); 2 Unidad de Tecnología de Alimentos, Secretaría de Investigación y Posgrado, Universidad Autónoma de Nayarit, Ciudad de la Cultura S/N, Colonia Centro, Tepic C.P. 63000, Nayarit, Mexico; guillermo.berumen@uan.edu.mx; 3 Centro de Investigaciones Biológicas del Noroeste, Km 1 Carretera a San Juan de La Costa “El Comitan”, La Paz C.P. 23205, Baja California Sur, Mexico; paola.magallon@gmail.com; 4 Bashan Institure of Sciences, 1730 Post Oak Ct, Auburn, AL 36830, USA; 5 Facultad de Química, Universidad Autónoma de Querétaro, C.U., Cerro de las Campanas S/N, Querétaro C.P. 76010, Querétaro, Mexico; iza.perez@hotmail.com; 6 Unidad Académica de Agricultura, Universidad Autónoma de Nayarit, Km 9 Carretera Tepic-Compostela, Xalisco C.P. 63780, Nayarit, Mexico; graciela.lopez@uan.edu.mx

**Keywords:** prebiotic, *Lactobacillus*, polyphenol, probiotic, growth promoters, soursop leaves

## Abstract

Soursop leaves are a source of phytochemical compounds, such as phenolic acids, flavonoids, hydrolyzable tannins, and acetogenins. These compounds can have several types of biological activities. Lactic acid bacteria can uptake phenolic compounds present in plants or fruits. The aim of the present work was to investigate the *in vitro* effect of hexane, acetone, methanolic, and aqueous extracts of soursop leaves (*Annona muricata* L.) on the growth, motility, and biofilm formation of *Lactobacillus casei*, and to determine compounds related to growth. The minimum concentration promoting growth, motility (swimming, swarming, and twitching), and biofilm-forming capacity (crystal violet) were evaluated. The results showed the growth-promoting capacity of acetone and aqueous extracts at low doses 25–50 mg/L, and an inhibition in the four extracts at higher doses of 100 mg/L. The *L. casei* growth is related to ellagic acid, quercetin rhamnoside, kaempferol dihexoside, quercetin hexoside, secoisolariciresinol, and kaempferol hexoside-rhamnoside. Hexane extract increased the three types of motility, while aqueous maintained swimming and twitching motility similar to control. The four extracts inhibited the biofilm formation capacity.

## 1. Introduction

The growing demand for functional foods and nutraceuticals has guided researchers to develop new products that meet the needs of consumers. An example of this is the use of beneficial bacteria, which can restore the balance of microbiota, stimulate the immune system, reduce digestive disorders, improve the absorption of nutrients, produce vitamins such as B and K, and prevent diseases [1,2]. Lactic acid bacteria (LAB) are a group of probiotic bacteria commonly used in the pharmaceutical and food industries [3], due to their low energy cost of production, production of compounds, such as lactic acid, flavorings, thickeners, and bacteriocins [4].

One of the most widely studied lactic acid bacteria, for its health-promoting properties, is *Lactobacillus casei*, a probiotic bacterium used to treat or prevent diverse diseases, so it is extensively used in the industry [5].

The demand for probiotics, such as *L. casei*, is increasing rapidly due to their impact on consumers’ health, so satisfying demand is a challenge [6]. Additionally, extracellular metabolic by-products of *L. casei* have been used as antagonist bacteria, bio-preservative, production of bacteriocin, and enzymes as clarification of juice [7,8,9,10].

Due to the above, recent research has focused on searching for new precursor resources for the growth of these microorganisms and the development of non-dairy products that preserve their viability and bioavailability [11,12].

Lactic acid bacteria can adapt to the characteristics of raw materials, such as plants or fruits, which are an abundant source of phenolic compounds [13]. Therefore, various interactions of medicinal plants and spices have been evaluated, such as extracts of oregano, pomegranate peel, and cloves, among others, which are rich in polyphenols. These plant materials have shown an antibacterial capacity against pathogenic bacteria at concentrations greater than 2500 µg/mL. However, they do not inhibit the growth of lactic acid bacteria (LAB) or probiotic bacteria [14]. Sutherland et al. [15] reported the growth of *Lactobacillus reuteri* by using aqueous extracts of garlic and black pepper. Furthermore, the aqueous extracts of banana, apple, and orange significantly increased the growth of *L. reuteri*, *L. rhamnosus*, and *Bifidobacteria lactis*. Park et al. [16] reported the production of lactic acid bacteria in fermented kimchi (cabbage, garlic, red pepper, and ginger).

Due to its wide nutraceutical and therapeutic use, a potential substrate for probiotics is soursop (*Annona muricata* L.) leaves [17]. It has bioactive properties, such as anti-inflammatory, anticarcinogenic, antidiabetic, antifungal, antibacterial, anthelmintic, and antiviral [18,19]. Furthermore, the soursop leaves are a source of antioxidant compounds and possess a wide diversity of phytochemical compounds, such as phenolic acids, flavonoids, hydrolyzable tannins, saponins, terpenoids, coumarins, annonaceous acetogenins, and cyclic hexapeptides [20,21]. The above compounds could be taken up by probiotic bacteria since their enzymes intervene in the glycosylation of the compounds, some oxidation processes, demethylation, and catabolism of small phenolic acids and aromatic compounds [22].

Therefore, this work aimed to investigate the in vitro effect of hexane, acetone, methanolic, and aqueous extracts of soursop leaves (*Annona muricata* L.) on the growth of *Lactobacillus casei*, and to determine compounds related to it. Further, we evaluate the effect of the extracts on the bacteria’s motility and biofilm-forming capacity.

## 2. Results

### 2.1. Effect of Extracts on Lactobacillus Casei Growth

**Figure 1:**
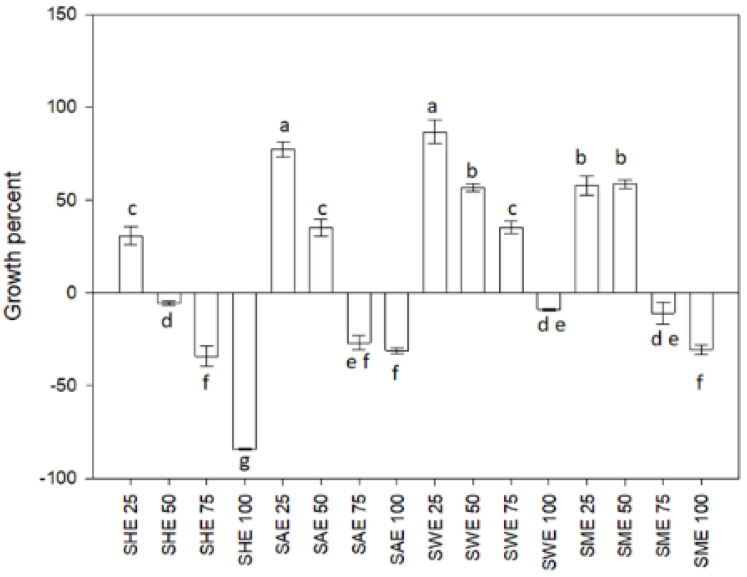
Growth percent of *Lactobacillus casei*. SHE soursop hexane extract, SAE soursop acetone extract, SME soursop methanol extract, and SWE soursop aqueous extract. Different letters indicate significant differences according to the Fisher LSD test (*p* < 0.05).

Figure 1 shows the effect of the dosage of soursop leaf extracts, SHE (hexane extract), SAE (acetone extract), SME (methanolic extract), and SWE (aqueous extract), on the growth of *L. casei*. SAE and SWE treatments at 25 µg/L showed the highest growth (*p* < 0.05); they increased the growth 80% above the control. However, all extracts at 100 µg/mL decreased the bacterium’s growth percentage.

### 2.2. Polyphenols in Soursop Leaf

Table 1 shows the content of polyphenolic compounds identified in the soursop leaf extracts. A total of 31 compounds were identified, including two flavanols, 12 flavonols, two hydroxybenzoic acids, 13 hydroxycinnamic acids, and two lignans. Of the identified compounds, 58% were found in the SAE, while SME presented 54% of the compounds. The polyphenols with the highest concentration were kaempferol dihexoside (286.01 µg/g) and kaempferol hexoside-rhamnoside (199.30 µg/g). Both compounds were found in SME.

### 2.3. Effect of the Polyphenols on the Growth of L. casei

**Figure 2:**
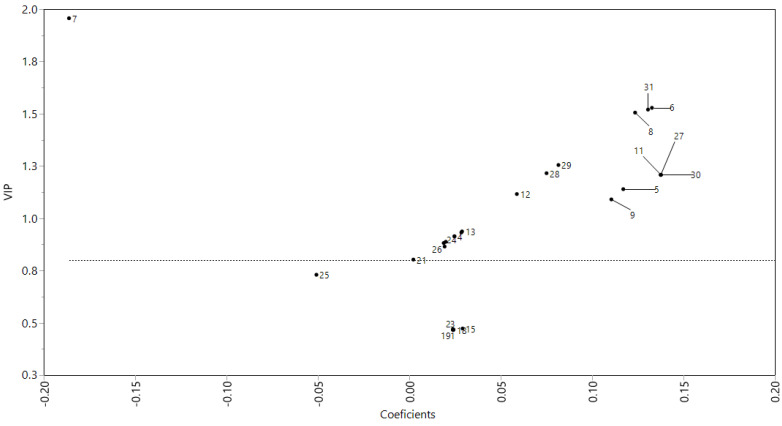
Association of polyphenols profile of soursop (*Annona muricata* L.) leaf with their effect on the *Lactobacillus casei* growth capacity through a PLS-DA model. PLS-DA, partial least square-discriminant analysis.

According to the PLS-DA model plot constructed with the profile of polyphenols and microbial growth (Figure 2), eight compounds from soursop leaf extracts were related to the *L. casei* growth (VIP > 0.8 and coefficients > 0.10). These compounds were identified as ellagic acid (27), quercetin rhamnoside (11), rhamnetin rhamnoside (8), coumaroyl hexoside (30), kaempferol dihexoside (5), quercetin hexoside (6), secoisolariciresinol (31), and kaempferol hexoside-rhamnoside (9). Quercetin hexoside (6), ellagic acid (27), coumaroyl hexoside (30), and secoisolariciresinol (31) were presented in the aqueous extract (SWE). These compounds are polar, so they have a greater affinity for water. Furthermore, quercetin hexoside was presented in acetone (SAE) and hexane (SHE) extracts, and secoisolariciresinol in hexane extract (SAE). Quercetin hexoside and secoisolariciresinol showed affinity for solvents of a range from polar to nonpolar, so they dissolve in water (polar), acetone (moderately polar), and, specifically, quercetin hexoside was soluble in hexane (nonpolar).

### 2.4. Effect of Extracts on Motility and Biofilm-Forming Capacity

**Figure 3:**
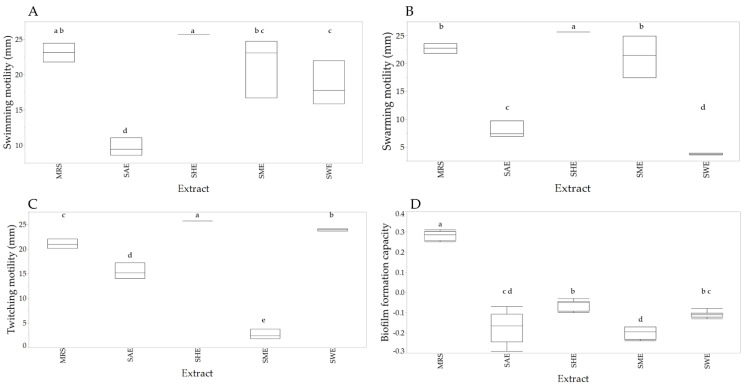
Box-plot of the effect of the extracts on displacement swimming (**A**), swarming (**B**), twitching (**C**), and biofilm-forming capacity (**D**). Different letters indicate significant differences according to the Fisher LSD test (*p* < 0.05).

According to bacterial growth results, the dose of 25 µg/mL was used to evaluate motility and biofilm formation capacity. Figure 3A shows the swimming-type displacement of *L. casei*. Treatment SAE decreased the bacterium displacement, while SHE increased the displacement regarding control (*p* < 0.05). On the other hand, SME and SWE did not show significant differences regarding control (*p* > 0.05).

Figure 3B shows that treatments SAE and SWE decreased the displacement swarming-type regarding control, while SHE increased the displacement (*p* < 0.05). SME did not show significant differences concerning control (*p* > 0.05). The displacement twitching of *L. casei* is shown in Figure 3C. Treatments SHE and SWE significantly increased this type of displacement, while SME and SAE decreased it compared to the control (*p* < 0.05). All extracts decreased the biofilm-forming capacity (*p* < 0.05) of *L. casei* (Figure 3D).

## 3. Discussion

Different properties have been found in soursop (*A. muricata* L.), such as antimicrobial, anti-inflammatory, antiprotozoal, antioxidant, insecticidal, larvicidal, and cytotoxic activity to tumor cells. These properties have been related to the more than 200 chemical compounds found in this plant [23]. In this study, 31 compounds were identified. Secoisolariciresinol (Sccl) and medioresinol (Mdl) have not been previously reported in soursop leaves. Secoisolariciresinol has been reported in pulp and seed of another species of the same genera, such as *Annona cherimola* [24].

The highest concentration of phenolic compounds was found in the methanolic extract, followed by water. Previous studies have shown that methanol has a greater affinity for phenolic compounds [25,26]. Phenolic compounds such as quercetin and its derivates present several hydroxyl groups, which gives it the hydrophilic character; however, they can be both lipo- and hydrophilic, depending if the molecule shows *O-*methyl, *C*-methyl, and phenyl derivates, which are lipophilic, and they could be solubilized in acetone or hexane [27]. The affinity of the solvents for different compounds could cause the extracts to have different biological activities.

The ability to affect microbial growth is one of the biological properties of plant extracts, either by inhibiting or increasing microbial growth. In this sense, we found a differentiated response depending on the dose. The lower evaluated concentrations of acetonic, methanolic, and aqueous extracts increased bacterial growth regarding control. The acetonic and aqueous at 25 µg/mL highlight increased microbial growth. On the other hand, we also found that the extracts at a dose of 100 µg/mL decreased bacterial growth. Some authors have reported that soursop leaf extract inhibited the growth of *Acanthamoeba triangularis*, *S. aureus*, *B. subtilis*, *E. coli*, *K. pneumonia*, and *Proteus vulgaris* using doses between 500 and 1000 μg/mL [28,29,30,31]. This behavior related to dose-response could be due to the phenomenon of hormesis. According to toxicology, hormesis is the effect of non-nutritional substances, which may have beneficial or stimulatory effects at low doses and produce adverse effects at higher doses [32,33]. Furthermore, Laparra and Sanz [34] mention that phytochemicals may inhibit pathogenic bacteria while stimulating the growth of beneficial bacteria, exerting prebiotic-like effects.

The stimulatory effect of acetone and aqueous extracts on the *L. casei* growth could be related to extracts composition. The previous could be because this bacterium has been characterized as an auxotrophic microorganism; namely, it cannot synthesize all growth factors, so it is necessary to obtain them from the growth medium [35]. Lee and Paik [36] mention that Lactic Acid Bacteria (LAB), as *L. casei*, use polyphenols and polysaccharides as substrates in fermentations.

The PLS-DA model contructed with the polyphenols profile of soursop leaves extracts and the bacterial growth showed that the main polyphenols associated with *L. casei* growth were ellagic acid (27), quercetin rhamnoside (11), rhamnetin rhamnoside (8), coumaroyl hexoside (30), kaempferol dihexoside (5), quercetin hexoside (6), secoisolariciresinol (31), and kaempferol hexoside-rhamnoside (9). These compounds showed the higher values of VIP (>0.8) and coefficients (>0.10), as we observe in Figure 2, which indicate that those compounds can be considered as the principal compounds associated with *L. casei* growth. Some authors mention that *Lactobacillus* spp. may metabolize polyphenols using bacterial enzymes, such as β-glucosidase, α-rhamnosidase, and β-glucuronidase. These enzymes could hydrolyze conjugated polyphenols and release glycosides and aglycones (flavonols as kaempferol, quercetin, quercitrin, and rutin, which are identified as aglycones) [36,37,38].

LAB can uptake oligosaccharides from the conjugated polyphenols and assimilate them through the fermentative metabolism of hexoses and pentose. This process can release aglycones that possess bioactive capacities (biological activity) as an antioxidant, antibacterial, preservative, and chemoprotective, among other properties [39].

Kaempferol and glycosylated derivates can protect against reactive oxygen species in cells [40]. In addition, these flavonoids can protect cells from different insults that lead to mitochondria-mediated cell death and attenuate oxidative stress and mitochondrial dysfunction [40,41].

Valero-Cases et al. [42] mention that lactic acid bacteria can uptake ellagic acid and increase survival. *Lactobacillus* spp. Can metabolize ellagic acid and secoisolariciresinol and biotransform them into secondary metabolites like enterodiol and enterolactone, according to Bravo et al., which give them one of their probiotic features. Furthermore, ellagic acid has been shown to prevent possible modifications to the mitochondrial membrane, oxidative stress, and toxins’ effects on cell division [43,44,45].

Regarding quercetin and its glycosylated derivates, Curiel et al. [46] found that quercetin has effects dependent on pH and compound concentration. Moreover, quercetin increased the growth and sugar consumption of *L. plantarum*. Braga et al. [47] asseverate that quercetin could prevent lipid oxidation by its antioxidant properties. This could help to maintain the membrane integrity on *L. casei*. Besides, Herranz-López et al. [48] assert that quercetin and quercetin-3-O-glucuronide can restore the mitochondrial mass and biogenesis, postulating that they could act as agonists of specific proteins. Finally, Lacerda et al. [49] mentioned that substances like quercetin and kaempferol in *Myrciaria jaboticaba* fruit extracts stimulated the growth and metabolic activity of probiotics such as *L. acidophilus*, *L. casei*, and *Bifidobacterium animalis* subsp. Lactis.

Moreover, the flavonol rhamnetin and hydroxycinnamic acids as coumaroyl hexoside from *Aloe arborescens* and *A. barbadensis* allowed the growth of lactic acid bacteria such as *Lactobacillus* [50]. According to Hervert-Hernández et al. [51], LAB can metabolize hydroxycinnamic acids such as coumaroyl hexoside by reducing the side chain to produce the corresponding 2-hydroxyphenylpropionic acids, which can then be descarboxylated to p-ethylphenols, allowing these bacteria to grow and modulate the functional composition of gut microbiota [52].

Furthermore, in order to grow, bacteria require nutrients, so they have strategies to obtain them. Motility is one of the ways bacteria can take nutrients to reach new niches for colonization [53]. *Lactobacillus casei*’s motility is poorly characterized [54]. Our results showed displacement swimming and swarming of *L. casei* exposed to SHE and SME at low concentrations.

Swimming and swarming motility are related to flagellar motility, however, *L. casei* does not present flagella, so its motility is related to pilus. In addition, the twitching motility is related to pilus. Sengupta et al. [55] reported the presence of sortase-mediated pilus gene clusters in many strains of *L. casei*.

Ellagic acid has shown antiquorum sensing activity related to swimming motility and biofilm-forming capacity [56]. It is natural for microorganisms to adhere to biotic or abiotic surfaces, multiply and become embedded in a viscous matrix [57]. However, the extracts evaluated in this work decreased the biofilm-forming capacity. The food industry’s particular concern is the maintenance of the microbial planktonic lifestyle through an efficient cleaning and production process. The formation and growth of biofilms are influenced by several factors, such as bacterial strain, surface and environmental properties, pH, nutrient concentration, and temperature [58]. Even today, four strategies are known to combat biofilms. One of them is the biochemical strategy to disrupt the biofilm using hydrolytic enzymes that degrade extracellular matrix components and the use of compounds able to bind or block intracellular communication, decreasing its level and promoting biofilm dispersion. Although the mechanism of how polyphenols affect the motility, adhesion, and biofilm formation of bacteria is not yet clear and can be very complex, the interaction of these with the protein-membrane and the activity of the quorum sensing has been established [58,59,60].

Altogether, our results showed the capacity of soursop leaf extracts as a potential prebiotic. These extracts are constituted for non-digestible compounds that beneficial microorganisms can metabolize, allowing to improve the bioavailability and bioactivity of the compounds. These compounds can increase the growth of *Lactobacillus casei*, which is known as a probiotic. These results suggest a wide application of extracts improving the biomass production and viability of probiotic bacteria as *L. casei*, into the health, pharmaceutical, food, agricultural, veterinary, and zootechnical industries.

## 4. Materials and Methods

### 4.1. Biologic Material

Mature soursop leaves (completely developed) were collected from the community of Lima de Abajo, Compostela, Nayarit, Mexico (21°56′7.1808″ N 105°15′28.584″ W), in August, 2018. Leaves were washed, disinfected, and shade dried at 30 °C for 15 days. These were ground in a food processor (Nutribullet^®^ NB-201, Ningbo, China) and stored in trilaminate bags at 25 °C until use. A commercial strain of *Lactobacillus casei* Shirota (Yakult ^®^, Guadalajara, Mexico) was used.

### 4.2. Preparation of Plant Extracts

The leaf powder was suspended in the extraction solvents (hexane, acetone, methanol, and water) at a ratio of 1:10. Subsequently, the suspensions were sonicated for 30 min at 42 Hz in an ultrasonic bath (BRANSON^®^ 5510, Danbury, CT, USA) [61]. The extracts were filtered on Whatman No. 1 paper using vacuum filtration equipment (20 Torr). The filtrates were concentrated on a rotatory evaporator (BÜCHI Labortechnik AG, CH) at 40 °C and 40 Torr vacuum. Finally, a stream of nitrogen gas was circulated over the extracts to remove residual solvent. The extracts were stored in an amber flask until their analysis. The extracts were labeled as hexane extract (SHE), acetonic extract (SAE), methanolic extract (SME), and aqueous extract (SWE).

### 4.3. Effect of Extracts on the Growth of Lactobacillus casei

#### 4.3.1. Inoculum Preparation

*Lactobacillus casei* was activated in 10 mL of Man, Ragosa, and Sharpe (MRS) medium for 24 h at 37 °C. Then, 1 mL aliquot was taken and added to 9 mL of MRS medium to prepare an inoculum; after, it was incubated for 16 h at 37 °C. Next, the inoculum was concentrated by centrifugation at 10,433× *g* for 10 min. Finally, a cellular suspension was prepared with an absorbance of 0.4 at a wavelength of 620 nm using a spectrophotometer (Thermo Fisher Scientific, *Multiskan* Go, Vantaa, Finland), equivalent to 10^8^ cells/mL [62].

#### 4.3.2. Determination of Minimum Growth-Promoting Concentration

The microdilution technique was used to determine the minimum growth-promoting concentration. Initially, a stock solution of the extracts was prepared in dimethyl sulfoxide (DMSO) at a concentration of 30,000 µg/mL. Then, four concentrations, 25, 50, 75, and 100 µg/mL, were prepared. Each treatment consisted of 175 µL of MRS medium, 20 µL of inoculum, and 5 µL of the extract [63]. The treatments were added to a sterile 96-well plate and incubated at 37 °C in a microplate reader (Thermo Fisher scientific, *MultiSkan* GO, Vantaa, Finland). The microplate was shaken every 5 min for 30 s. The OD at 620 nm was read after 24 h of incubation. The growth percentage was determined using the Equation (1).

% Growth = (OD initial − OD sample)/(OD control) × 100(1)

### 4.4. Phytochemical Analysis

First, the polyphenols were extracted using the method described by Arranz et al. [64]. Then, 2 mL of the polyphenol extract (EPF) were concentrated in a 16,000× *g* vacuum centrifuge for 10 min at 4 °C. Next, the concentrate was resuspended in 200 µL of methanol and filtered using a PVDF syringe filter (13 mm, 0.45 µm) and stored in microvials until analysis.

The phytochemical profile was assessed in an Ultra-Performance Liquid Chromatograph (UPLC) coupled to a Diode Array Detector (DAD) and a Quadrupole Time-of-Flight (Q-ToF) mass spectrometer (MS) with an electrospray ionization (ESI) interphase (Vion IMS, Waters Co., Milford, MA, USA). Decoctions were filtered (0.45 mm) and directly injected into a BEH Acquity C18 column (2.1 × 100 mm, 1.7 mm) at 35 °C. For the chromatographic separation, water with 0.1% formic acid (A) and acetonitrile (B) were used as mobile phase at a flow of 0.5 mL/min. The gradient conditions were 0% B/0 min, 15% B/2.5 min, 21% B/10 min, 90% B/12 min, 95% B/13 min, 0% B/15 min, and 0% B/17 min. Absorbances were measured at 214, 280, 320, 360, 484, and 535 nm [65]. The following commercial standards were used to construct calibration curves and quantitate the different types of phenolic compounds: (+)-catechin (flavanols), naringenin (flavanones), quercetin (flavonols), p-hydroxybenzoic acid (hydroxybenzoic acids), and chlorogenic acid (hydroxycinnamic acids). Polyphenol results are expressed as µg/g of extract, whereas lignan and stilbene results are expressed as arbitrary units. The following MS conditions were used: capillary voltage, 2.0 kV; cone voltage, 40 eV; low collision energy, 6 V; high collision energy, 15–45 V; source temperature, 120 °C; cone gas flow, 50 L/h; and desolvation gas, N2 at 450 °C and 800 L/h. Data acquisition was carried out at negative ionization mode (ESI-) within a 100–1200 Da mass range. Leucine-enkephalin solution (50 pg/mL) was used for lock mass correction at 10 mL/min. Identification was carried out by analysis of the exact mass of the pseudomolecular ion (mass error < 5 µg/mL), isotope distribution, and fragmentation pattern.

### 4.5. Bacterial Motility

Bacterial motility (swimming, swarming, and twitching) was determined by measuring the displacement (mm) of the bacterium in MRS medium supplemented with agar. The medium was supplemented with 0.3% agar to swimming-type motility, 0.6% agar to swarming-type motility, and 1% agar to twitching, as Burrows [66] described. A total of 25 µg/mL of each soursop leaf extract was added to the culture media in each technique. The percentage displacement in each technique was determined using the Equation (2).

(2) $$\%\mathrm{Displacement}=\frac{\mathrm{Displacement}\;\mathrm{with}\;\mathrm{extract}}{\mathrm{Displacement}\;\mathrm{in}\;\mathrm{control}\;\mathrm{treatment}}\times 100\%$$

### 4.6. Biofilm Forming Capacity

The method proposed by Naves et al. [67] was followed with slight modifications. First, 200 µL of MRS medium at 50%, 10 µL of inoculum, and 25 µL of extract at 25 µg/mL were added to 96-well microplates. The cultures were incubated at 37 °C for 18 h. After that, OD at 620 nm was read in a microplate reader (Thermo Fisher scientific, MultiSkan GO, Vantaa, Finland). Then, the plate was washed with saline solution to remove unattached bacteria. Next, the plate was dried under airflow for 20 min. Wells were stained with 200 µL of crystal violet at 0.3%, washed with distilled water, and dried for 1 h. After, wells were filled with 200 µL of 95% ethyl alcohol, and absorbance was read at 540 nm. The biofilm-forming capacity (BFC) was calculated with Equation (3).

(3) $$\mathrm{BFC}=\frac{\mathrm{AB}-\mathrm{CW}}{G}$$

*AB* is the DO540 nm of the adhered bacterium to the microplate, *CW* is the DO540 of the control medium (MRS without bacteria), *G* is the DO620 nm of cell growth.

### 4.7. Statistical Analysis

Each extract was analysed by triplicate in three independent experiments. A completely randomized design was used. The data obtained in the microdilution technique to determine the bacterium minimum growth-promoting concentration of the bacterium were analysed by ANOVA with α = 0.05 to determine significant differences in the extracts. An LSD Fisher test (α = 0.05) was used when ANOVA showed statistical differences.

The association between polyphenolic compounds and the bacterial growth were analysed by plots means of variable importance in projection (VIP) vs. coefficient, constructed from partial least squares-discriminant analysis (PLS-DA) with centered and scaled data. A nonlinear intervention in partial least squares (NIPALS) was used. All analyses were carried out with JMP software 14.3 (Sytat Software, Inc., San José, CA, USA).

## 5. Conclusions

The acetone, methanolic, and aqueous extracts of soursop at 25 µg/mL increased the bacterial growth. However, all evaluated extracts inhibited the bacterial growth at 100 µg/mL. Hexane and methanolic extract of soursop leaf did not show differences with respect to control on swimming motility, while aqueous and acetonic decreased it. Hexane extract increased the swarming motility, methanolic extract maintained it, while acetone and aqueous extracts decreased this motility concerning control. Hexane and aqueous extracts increased twitching motility, while acetone and methanolic extracts decreased this type of motility. All extracts decreased the biofilm-forming capacity of *Lactobacillus casei*.

In order to obtain higher *L. casei* growth and to use the less toxic solvent, the best treatment was the aqueous extract at 25 µg/mL, which increased the bacterial growth and maintained the swimming and twitching motility without differences regarding control.

The *L. casei* growth was related to ellagic acid, quercetin rhamnoside, kaempferol dihexoside, quercetin hexoside, secoisolariciresinol, and kaempferol hexoside-rhamnoside. 

The study suggests a broad range of applications to the aqueous extract of soursop leaf into the health, pharmaceutical, food, agricultural, veterinary, and zootechnical industries since it improves the biomass production and viability of probiotic bacteria as *L. casei*.

## Figures and Tables

**Table 1:** Polyphenols profile of extracts from soursop (*Annona muricata* L.).

Family	Code	Compound	Concentration (mg/g)			
			SAE	SHE	SME	SWE
Flavanols	1	(+)-Catechin *	ND	ND	5.96 ± 0.41	ND
	2	(-)-Epicatechin *	ND	ND	27.15 ± 1.53	ND
Flavonols	3	Quercetin dihexoside	ND	ND	0.06 ± 0.00	ND
	4	Quercetin rutinoside (rutin) *	0.02 ± 0.00	ND	ND	ND
	5	Kaempferol dihexoside	10.03 ± 1.11	0.02 ± 0.00	286 ± 44.07	185.82 ± 8.31
	6	Quercetin hexoside	27.04 ± 2.58	0.03 ± 0.00	ND	23.42 ± 1.30
	7	Quercetin xylosyde	ND	0.02 ± 0.00	ND	ND
	8	(Iso)-rhamnetin rhamnoside	0.39 ± 0.03	ND	0.35 ± 0.03	0.19 ± 0.00
	9	Kaempferol hexoside-rhamnoside	9.17 ± 1.06	0.01 ± 0.00	199.30 ± 14.59	115.94 ± 18.51
	10	Kaempferol hexoside	32.30 ± 3.21	ND	ND	ND
	11	Quercetin rhamnoside	ND	ND	0.03 ± 0.00	60.38 ± 6.48
	12	Quercetin *	15.21 ± 0.71	0.06 ± 0.00	ND	3.51 ± 0.21
	13	Kaempferol *	21.01 ± 1.29	0.27 ± 0.01	ND	0.92 ± 0.06
	14	(Iso)-rhamnetin	0.40 ± 0.03	0.01 ± 0.00	ND	ND
Hydroxy-		Dihydroxybenzoic acid hexoside	5.41 ± 0.22	ND	33.43 ± 1.46	ND
benzoic	15					
acids						
	16	Hydroxybenzoic acid *	ND	ND	0.20 ± 0.01	ND
Hydroxy-	17	Caffeoylquinic acid isomer I *	ND	ND	15.70 ± 0.89	ND
cinnamic acids			ND	ND		ND
	18	Coumaroylquinic acid isomer I	ND	ND	11.88 ± 0.68	ND
	19	Caffeoylquinic acid isomer II	ND	ND	6.20 ± 0.33	ND
	20	Sinapic acid hexoside	0.78 ± 0.06	ND	0.09 ± 0.01	ND
	21	Feruloylquinic acid isomer I	0.32 ± 0.02	0.039 ± 0.00	ND	ND
	22	Coumaroylquinic acid isomer II	ND	ND	17.00 ± 0.91	ND
	23	Caffeoylquinic acid isomer III	ND	ND	ND	ND
	24	Feruloylquinic acid isomer II	0.55 ± 0.04	0.039 ± 0.00	0.14 ± 0.01	ND
	25	Coumaric acid *	ND	0.023 ± 0.00	0.06 ± 0.00	ND
	26	Feruloylquinic acid isomer III	0.62 ± 0.04	0.02 ± 0.00	ND	ND
	27	Ellagic acid *	ND	ND	ND	0.07 ± 0.01
	28	Ferulic acid *	0.74 ± 0.03	ND	ND	0.25 ± 0.01
	29	Sinapic acid *	0.15 ± 0.01	ND	ND	0.06 ± 0.00
	30	Coumaroyl hexoside	ND	ND	ND	0.08 ± 0.00
		Total	124.142 ± 10.45	0.121 ± 0.00	603.54 ± 64.93	204.82 ± 26.58
Family		Compound		Response (AU)		
			SAE	SHE	SME	SWE
Lignanes	31	Secoisolariciresinol	4651.90 ± 440.68	ND	ND	3877.30 ± 147.70
	32	Medioresinol	ND	ND	2645.60 ± 290.4	ND

* Identification confirmed with commercial standards. (ND) not detected.

## Data Availability

The data used to support the findings of this study are available from the corresponding author upon request.
